# Prevalence of stunting and overweight/obesity among Brazilian children according to different epidemiological scenarios: systematic review and meta-analysis: Prevalências de déficit de estatura e de sobrepeso/obesidade em crianças brasileiras, segundo diferentes cenários epidemiológicos: revisão sistemática com metanálise

**DOI:** 10.1590/1516-3180.2015.0227121

**Published:** 2015-04-14

**Authors:** Carolina Pereira da Cunha Sousa, Ricardo Alves de Olinda, Dixis Figueroa Pedraza

**Affiliations:** 1 MSc. Postgraduate Public Health Program, Universidade Estadual da Paraíba (UEPB), Campina Grande, PB, Brasil.; 2 PhD. Professor in the Department of Statistics, Universidade Estadual da Paraíba (UEPB), Campina Grande, PB, Brasil.; 3 PhD. Professor in the Department of Nursing and Postgraduate Public Health Program, Universidade Estadual da Paraíba (UEPB), Campina Grande, PB, Brasil.

**Keywords:** Child, Body height, Growth, Overweight, Obesity

## Abstract

**CONTEXT AND OBJECTIVE:**

Within the Brazilian nutritional panorama, coexistence of antagonistic nutritional disorders can be seen, especially the increasing prevalence of overweight and the persistence of significant rates of chronic malnutrition in vulnerable groups of the population. Because these are major public health problems, this study aimed to ascertain the prevalence of stunting and overweight/obesity among Brazilian children according to different epidemiological scenarios.

**DESIGN AND SETTING:**

This was a systematic review of prevalence studies, developed at the State University of Paraíba.

**METHODS:**

The SciELO, Lilacs and PubMed databases were searched for articles, using specific keywords. Articles published between 2006 and 2014 were selected. The review was conducted by two reviewers who worked independently. A systematic review with meta-analysis was conducted, for which the studies were grouped within different epidemiological settings.

**RESULTS:**

Among the 33 articles recovered, 9 involved samples from daycare centers, 4 had samples from public healthcare services or social registers, 5 related to populations in situations of social inequity and 15 were population-based. Higher chances of stunting were found in populations in situations of social inequity and in those at public healthcare services or on social registers, in relation to reference populations. For overweight/obesity, none of the scenarios had a higher chance than the reference.

**CONCLUSION:**

Among Brazilian children, stunting continues to be a socially determined public health problem that mainly affects marginalized populations. This problem coexists with significant rates of overweight/obesity affecting all social groups.

## RESUMO

**CONTEXTO E OBJETIVO:**

O panorama nutricional brasileiro revela a coexistência de agravos nutricionais antagônicos, com destaque para as prevalências crescentes de excesso de peso e a persistência de taxas expressivas de desnutrição crônica em grupos vulneráveis da população. Por constituírem importantes problemas de saúde pública, este artigo teve por objetivo verificar as prevalências de déficit de estatura e de sobrepeso/obesidade em crianças brasileiras segundo diferentes cenários epidemiológicos.

**TIPO DE ESTUDO E LOCAL:**

Revisão sistemática de estudos de prevalência, desenvolvida na Universidade Estadual da Paraíba.

**MÉTODOS:**

Os artigos foram pesquisados nos bancos de dados SciELO, Lilacs e PubMed, usando-se palavras-chave específicas. Selecionaram-se artigos publicados entre 2006 e 2014. A revisão foi conduzida por dois revisores de forma independente. Realizou-se revisão sistemática com metanálise, para a qual os estudos foram agrupados em diferentes cenários epidemiológicos.

**RESULTADOS:**

Dentre os 33 artigos recuperados, 9 envolveram amostras obtidas em creches, 4 de serviços públicos de saúde ou de cadastros sociais, 5 foram referentes a populações com iniquidades sociais e 15 tinham base populacional. Maiores chances de déficit de estatura foram constatadas nas populações com iniquidade social e naquelas provenientes de serviços públicos de saúde ou de cadastros sociais, em relação às populações de referência. Para o sobrepeso/obesidade, nenhum cenário apresentou maior risco do que a referência.

**CONCLUSÕES:**

Nas crianças brasileiras, o déficit de estatura continua representando um problema de saúde pública de determinação social que atinge, principalmente, populações marginalizadas. Essa problemática coexiste com taxas expressivas de sobrepeso/obesidade que afetam todos os grupos sociais.

## INTRODUCTION

Within the current Brazilian nutritional panorama, a declining trend of malnutrition followed by a rapid increase in the prevalence of childhood overweight and obesity can be seen.^1^ These paradoxical data are explained by the process of nutritional transition, characterized by inversion of the distribution patterns of nutritional problems.^2^

It has been observed over recent years that the prevalence of overweight/obesity among Brazilian children exceeds the prevalence of weight deficit, with similar behavior in all regions of the country.^3^ The exponential increase in overweight/obese rates serves as a warning of the epidemic nature of this disease.^4^

On the other hand, stunting remains an alarming public health problem in Brazil. Children of low socioeconomic status are more vulnerable to this and its prevalence varies within and between regions.^5^^,^^6^^,^^7^ This context, with uneven advances in terms of nutritional status, indicates that the prevalence of chronic malnutrition in vulnerable groups remains high, especially among children in indigenous populations (26%) and *quilombo* populations (descendants of escapees from slavery; 16%), as well as among those who are beneficiaries of income transfer programs (15%). In relation to excess weight, rapid growth in all age and income groups has been observed.^3^

Stunting during childhood can have undesirable consequences such as increased incidence and severity of infectious diseases, increased infant mortality rates, delayed psychomotor development, lower school performance, emergence of chronic diseases and reduced production capacity in adulthood, with losses in economic growth and social development of the country. Female children of short stature present higher risk that, in adulthood, they will have children with low birth weight, which will have negative effects on nutritional status and morbidity and mortality.^8^

If present early in childhood, obesity is associated with development of chronic diseases in adulthood, decreased quality of life and high healthcare costs. Obesity early in life is a risk factor for respiratory problems, type 2 diabetes mellitus, hypertension, dyslipidemia, metabolic syndrome, atherosclerosis, acute myocardial infarction and stroke. In addition, it is related to psychosocial complications due to social withdrawal consequent to discrimination.^9^^,^^10^^,^^11^

## OBJECTIVE

Because of the importance that stunting still presents among children, as a public health problem coexisting with greater overweight/obesity, this study aimed to determine the prevalence of stunting and overweight/obesity among Brazilian children according to different epidemiological settings.

## METHODS

This was a systematic review with meta-analysis on scientific studies on stunting and overweight/obesity conducted in Brazil that included preschool children and/or those younger than five years of age. Articles of interest were identified by two reviewers who searched the SciELO, Lilacs and PubMed databases independently. The search was limited to title words or abstracts, using the following combination of descriptors in Portuguese and English respectively: (*estado nutricional/nutritional status* OR *crescimento/growth* OR *antropometria/anthropometry* OR *desnutrição/malnutrition* OR *déficit de estatura/stunting* OR *baixa estatura/short stature* OR *sobrepeso/overweight* OR *obesidade/obesity* ) AND (*criança/child* OR *pré-escolar/preschool children* ). 

Articles published between 2006 and the search date (February 26, 2015) were taken into consideration for the purpose of this review. We chose a period beginning in 2006 since this was the year of publication of the new growth curves adopted by the World Health Organization and the year of the last National Survey of Demographics and Health of Children and Women.^12^

All articles identified in the databases were entered into an Excel spreadsheet, with the objective of detecting repetitions of documents in the same database and duplicates in different databases. These procedures were used with the intention of ensuring greater accuracy and reliability of the review results. This spreadsheet was used later on to extract data from selected studies, and it brought together information about the author, publication date, study type, age of participants and outcomes of interest.

The criteria used for including articles in this review were that these needed to be observational studies with representative randomly selected samples; studies with descriptions of stunting prevalence estimated using the anthropometric index of height for age and/or overweight/obesity; studies with estimates using the anthropometric indexes of weight for height or body mass index for age; and studies including preschool Brazilian children and/or those younger than five years of age.

Regarding exclusion criteria, the following studies were considered to be unsuitable for the proposed objectives: review articles; intervention studies; theses; letters to the editor; editorials; correspondence; qualitative studies; studies including deaths; studies conducted outside of Brazil; studies from secondary data (subject to bias); studies based on secondary analysis on population-based nationwide data or specific populations (from which the results were already known and thus their use would diverge from the aim constructed here or would introduce bias); population-based studies on some kind of illness or preexisting nutritional deficit; studies that did not include preschool children and/or those under the age of five years; studies with unrepresentative samples and/or non-random selection (case studies, reports on experiences, clinical cases or case series); studies that lacked data on the outcomes of interest; studies that did not show the prevalence of stunting and/or overweight/obesity among children aged 0-60 months; studies using reference populations that differed from the ones used by the World Health Organization; and studies that did not report the cutoff points for the nutritional diagnosis or that used inadequate parameters.

The cutoff point that was considered to be appropriate for making a diagnosis of stunting was height for age < -2 or ≤ -2 Z-scores. For overweight/obesity, cutoff points for weight-for-height or body mass index for age > 2 or ≥ 2 Z-scores were considered appropriate. Although the cutoff points that are suitable for diagnosing stunting and overweight/obesity are height-for-age < -2 Z-scores and weight-for-height or body mass index for age > 2 Z-scores, respectively, values ≤ -2 Z-scores for stunting and ≥ 2 Z-scores for overweight/obesity were also considered adequate because these have been used by some authors.

Titles and abstracts were read in order to identify review articles, intervention studies, theses, letters to the editor, editorials, correspondence and qualitative studies. The method or the full study was read in order to identify other selection criteria.

Subsequently, the lists of references of articles already included in the review were reviewed in order to try to identify additional articles. Articles thus identified then underwent the same processes as used for articles that had earlier been identified in bibliographic databases, and were included in this review if it was possible to confirm compliance with the selection criteria.

Discrepancies between the reviewers in the literature search, study selection and classification of articles as included or excluded were resolved by reaching a consensus. This observation was also valid for articles identified in the list of references. Articles not fully available online were purchased.

The articles classified as meeting the selection criteria, and thus included in this review, were characterized according to the following parameters: source, study site, age group, sample size, anthropometric indexes and results (prevalence of stunting and/or overweight/obesity). Furthermore, the quality of the studies was assessed using the critical appraisal tool for prevalence studies that was developed and tested by Munn et al.^13^ This tool consists of 10 questions on the adequacy and accuracy of the study, relating to the validity of the methods, interpretation and applicability of the results. Each item was rated with one point when the answer was yes or not applicable, half a point when the answer was unclear and zero points when the answer was no, thus generating a maximum score of 10 points. The score for each article was used to classify the articles into three quality categories: 8 to 10 (high quality); 5 to 7 (average quality); and 0 to 4 (low quality).

The articles were also grouped into four categories according to where their respective samples came from: daycare centers; public healthcare services or social registers; populations in situations of social inequity; and population-based studies representing cities, regions or states. These categories represented epidemiological study scenarios.

In order to produce a synthesis of the data, the results from the studies were systematized by considering the variations in the prevalence of stunting and overweight/obesity according to the epidemiological scenarios adopted. For each scenario, the average prevalence weighted according to sample size and range was calculated. To make statistical syntheses on the first three scenarios, odds ratios for 95% confidence intervals regarding stunting and overweight/obesity were calculated, taking the prevalences found in the National Survey of Demographics and Health of Children and Women^12^ and in the population-based studies systematized in this review as the reference data. The significance of differences (P < 0.05) among the frequencies found was also ascertained using the chi-square test. The software used in statistical analyses was Rv2.10.0.

## RESULTS

**Figure 1:**
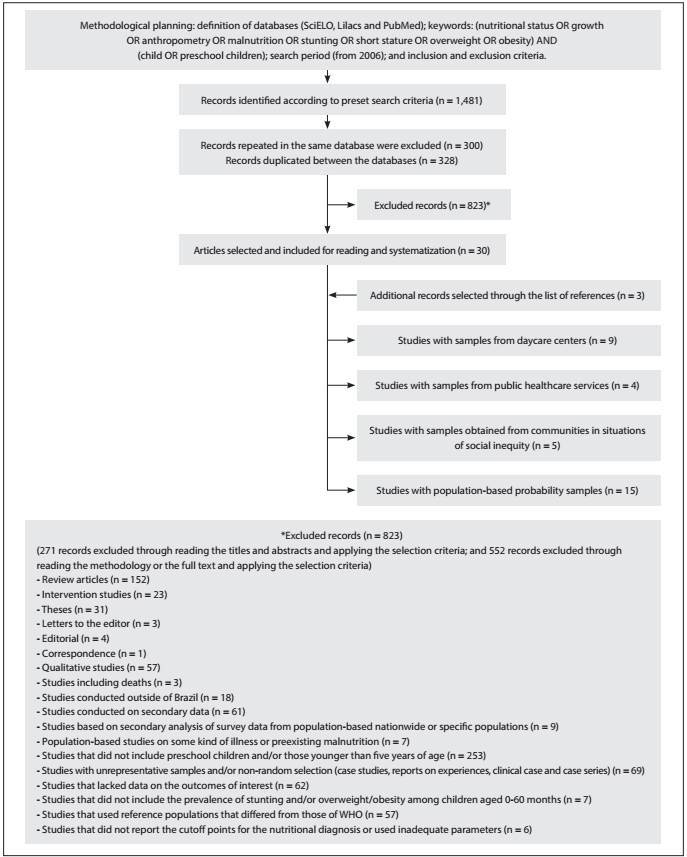
Flowchart used for identification and selection of studies on stunting and/or overweight/obesity among children that were conducted in Brazil and published between 2006 and 2014.

Through the above procedures, 1,481 records were identified: 402 in SciELO (of which 199 were repeated), 593 in Lilacs (of which 101 were repeated) and 486 in PubMed. A total of 328 records were replicated among the databases and, thus, 853 documents were identified, after excluding those that were repeated or duplicated. After applying the selection criteria, 823 documents were excluded and 30 articles were considered suitable for the study purposes. Consultation of the reference lists of these 30 articles produced another three articles that met the selection criteria, and therefore a total of 33 articles were read and systematized. Nine of these involved samples from daycare centers, four had samples from primary healthcare units or social registers, five had samples from populations in situations of social inequity and 15 used representative population-based samples from cities, regions and states in Brazil. Figure 1 shows the flowchart used for identification and selection of studies.

In assessing the quality of articles, six were categorized as medium quality and the other 24, as high quality. The quality criterion for which the articles showed greatest limitation was, remarkably, identification of confounding factors. Given that the estimated quality of all the articles was average or high and that the main risks of bias within the analysis related to confounding factors, without impairing the review of goals, it was decided to systematize all the studies.

### Prevalence of stunting and overweight/obesity in samples from daycare centers

Nine articles^14^^,^^15^^,^^16^^,^^17^^,^^18^^,^^19^^,^^20^^,^^21^^,^^22^ with sample sizes ranging from 189^19^ to 676^22^ were included. The prevalence of stunting and overweight/obesity ranged from 3.3%^21^ to 20.5%^19^ and from 2.3%^21^ to 7.5%,^15^ respectively. The average prevalence of stunting weighted for sample size was 9.11%, and the average prevalence of overweight/obesity weighted for sample size was 5.37% (Table 1).

**Table 1:**
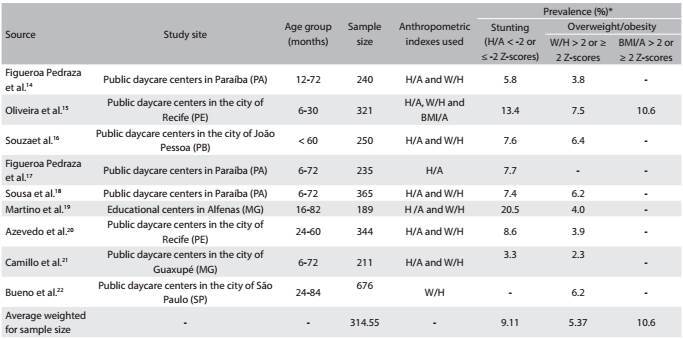
Prevalence of stunting and overweight/obesity, according to studies published between 2006 and 2014, involving samples taken from daycare centers located in Brazil

H/A = height-for-age; W/H = weight-for-height; BMI/A = body mass index for age. *Although the cutoff points suitable for making the diagnoses of stunting and overweight/obesity are the H/A values < -2 Z-scores and W/H or BMI/A > 2 Z-scores, respectively, values ≤ -2 Z-scores for stunting and ≥ 2 Z-scores for overweight/obesity were also considered adequate, because they have been used by some authors.

### Prevalence of stunting and overweight/obesity in samples obtained from primary healthcare units or social registers

Four articles^23^^,^^24^^,^^25^^,^^26^ involving sample sizes ranging from 155^23^ to 443^25^ children were included. Stunting was evaluated in only two studies and showed prevalence rates of 6.3%^25^ and 9.7%.^26^ According to the body mass index for age, overweight/obesity ranged from 5.2%^25^ to 17.9%.^24^ The average prevalence of stunting weighted for sample size was 7.25% for stunting and the average prevalence of overweight/obesity weighted for sample size was 10.97% (diagnosed using the body mass index for age) (Table 2).

**Table 2:**
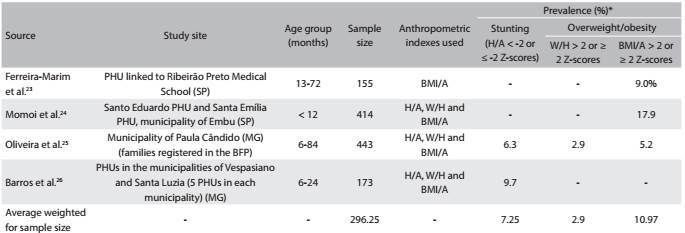
Prevalence of stunting and overweight/obesity among children, according to studies published between 2006 and 2014, involving samples from primary healthcare units or social registers in Brazil

PHU = primary healthcare unit; BFP = Bolsa Família program; H/A = height-for-age; W/H = weight-for-height; BMI/A = body mass index for age. *Although the cutoff points suitable for making the diagnoses of stunting and overweight/obesity are the H/A values < -2 Z-scores and W/H or BMI/A > 2 Z-scores, respectively, values ≤ -2 Z-scores for stunting and ≥ 2 Z-scores for overweight/obesity were also considered adequate, because they have been used by some authors.

### Prevalence of stunting and overweight/obesity in samples obtained from populations in situations of social inequity

Five articles^27^^,^^28^^,^^29^^,^^30^^,^^31^ involving sample sizes ranging from 99^27^ to 973^28^ children were included. For stunting, prevalences from 11.5%^28^ to 45.3%^29^ were found. For the diagnosis of overweight/obesity, all the studies used weight-for-height, while only two^29^^,^^30^ used the body mass index for age. The minimum and maximum prevalences were 2.1^29^ and 7.1%,^28^ according to weight-for-height, and they were 5.9%^29^ and 6.4%,^30^ according to the body mass index for age. The average prevalence of stunting weighted for sample size was 21.42% and the average prevalence of overweight/obesity weighted for sample size was 5.64% and 6.04%, respectively from the weight-for-height and body mass index for age indexes (Table 3).

**Table 3:**
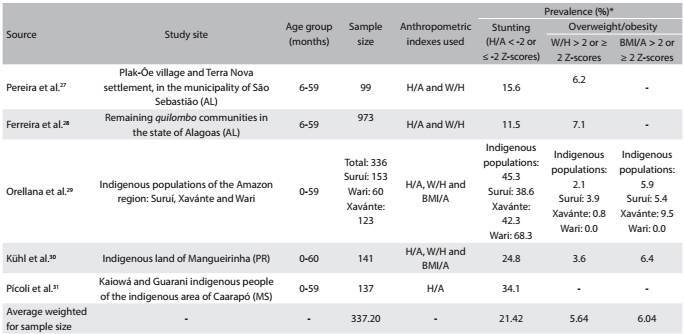
Prevalence of stunting and overweight/obesity among children, according to studies published between 2006 and 2014, involving samples obtained from populations in situations of social inequity located in Brazil

H/A = height-for-age; W/H = weight-for-height; BMI/A = body mass index for age. *Although the cutoff points suitable for making the diagnoses of stunting and overweight/obesity are the H/A values < -2 Z-scores and W/H or BMI/A > 2 Z-scores, respectively, values ≤ -2 Z-scores for stunting and ≥ 2 Z-scores for overweight/obesity were also considered adequate, because they have been used by some authors.

### Prevalence of stunting and overweight/obesity in studies representative of cities, regions or states of Brazil

Fifteen articles^32^^,^^33^^,^^34^^,^^35^^,^^36^^,^^37^^,^^38^^,^^39^^,^^40^^,^^41^^,^^42^^,^^43^^,^^44^^,^^45^^,^^46^ with sample sizes ranging from 164^37^ to 6,397^33^ children were included. The lowest prevalence of stunting found was 5.0%^32^ and the highest was 16.5%.^38^ The prevalence of overweight/obesity ranged from 3.2%^37^ to 12.5%^39^, according to weight-for-height and from 6.3%^38^ to 11.2%,^34^ according to the body mass index for age. The average prevalence of stunting weighted for sample size was 10.02%. For overweight/obesity, the weighted average prevalence was 10.18%, according to weight-for-height and 7.70%, according to the body mass index for age (Table 4).

**Table 4:**
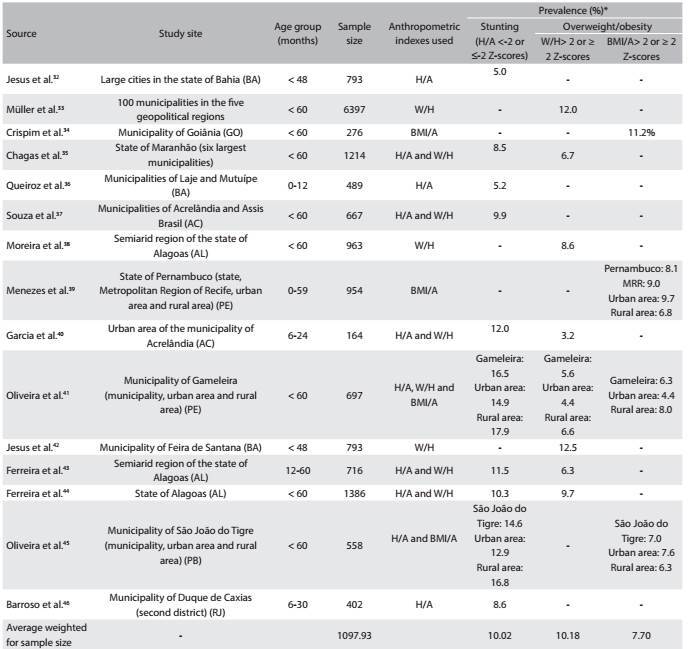
Prevalence of stunting and overweight/obesity among children, according to studies published between 2006 and 2014, involving samples representative of cities, regions or states in Brazil

H/A = height-for-age; W/H = weight-for-height; BMI/A = body mass index for age. *Although the cutoff points suitable for making the diagnoses of stunting and overweight/obesity are the H/A values < -2 Z-scores and W/H or BMI/A > 2 Z-scores, respectively, values ≤ -2 Z-scores for stunting and ≥ 2 Z-scores for overweight/obesity were also considered adequate, because they have been used by some authors.

### Prevalence of stunting and overweight/obesity among Brazilian children according to different epidemiological scenarios

Table 5 shows the results found for the analysis categories. It was observed that the highest prevalence of stunting was found among populations in situations of social inequity (21.42%). Taking the prevalence of stunting observed in population-based studies in cities, regions or states in Brazil (10.02%) as a reference, it was found that the likelihood that a child belonging to a population in a situation of social inequity would show stunting was 2.38 times greater (95% CI: 1.03-6.01). Samples from public healthcare services were also likely to show stunting: 2.37 (95% CI: 1.01-6.03). Similar findings were observed when the results from the National Survey of Demographics and Health of Children and Women were taken as the reference.^12^ The odds ratios described above were not observed in relation to children at daycare centers or samples from public healthcare services or social registers. With regard to overweight/obesity, it was observed that none of the categories under review (daycare centers, public healthcare units and social registers, and populations in situations of social inequity) had odds ratios higher than the outcome in relation to reference populations.

**Table 5:**
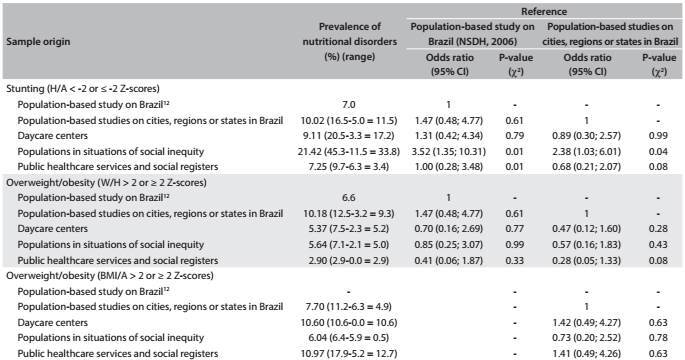
Odds ratios and 95% confidence intervals for stunting and overweight/obesity among Brazilian children according to different epidemiological scenarios considering the studies published between 2006 and 2014, taking the reference data to be the prevalence found in the NSDH^12^ and the population-based studies on cities, regions or states in Brazil

NSDH = National Survey of Demographics and Health of Children and Women; CI = confidence interval; H/A = height-for-age; W/H = weight-for-height; BMI/A = body mass index for age.

## DISCUSSION

While nationwide population surveys have shown prevalences of 6.0% of stunting among children younger than five years of age^47^ and 7.0%,^12^ similar works focusing on populations in situations of social inequity have shown prevalences of 15.0% in populations living in *quilombos* ^48^ and 25.7%^49^ in indigenous populations. In addition, temporal analyses have indicated that there have been sharp declines in the prevalence of stunting among Brazilian children.^50^^,^^51^ However, it is possible that different behavior has been conditioned, for example, by the length of time that individuals have been benefiting from income transfer programs.^52^ Analysis according to income group has indicated that the risk of childhood malnutrition in Brazil is strongly determined by family income.^43^ Moreover, analyses on the institutionalization of children in daycare centers have suggested that daycare attendance has a protective effect on children's growth.^53^^,^^54^ The results from this study showed that populations in situations of social inequity are more likely to present stunting than reference populations, unlike the results from studies in which the samples were from daycare centers.

Thus, it was observed that, despite evidence of reductions in the prevalence of stunting among children below the age of five years, this condition still remains a public health problem associated with social inequalities. These findings show that there is a need for measures to prevent stunting, including actions within the socioeconomic, health and educational spheres focusing on socially and economically vulnerable populations. In this regard, care provided through specific programs such as daycare centers and within the context of the Bolsa Família program (an income supplementation program), with assurances regarding the conditions attached to such programs, seems to offer continued assistance for protecting the nutritional status and health of vulnerable children.

The importance of daycare centers in relation to children's nutritional status comes from the fact that they are long-term care institutions that provide virtually all daily meals and constitute an ideal environment for implementation of health promotion strategies and actions, which are conditions that directly affect the nutritional status and growth of children.^2^^,^^53^ Thus, daycare centers are an important instrument for ensuring food and nutrition safety, and their role has gradually been expanding such that they are becoming a public policy proposal for the education, nutrition and health sectors.^15^^,^^20^

Despite the relevance of actions such as providing benefits through the Bolsa Família program, which has been a timely initiative for improving the living conditions of low-income families, it has been suggested that this action alone does not satisfactorily ensure adequate food and nutritional safety levels.^55^ This argument is justified with regard to determining children's growth from the perspective of social and economic inequality, as well as in relation to child health inequities. In this context, inadequate conditions within the social and economic environment produce deprivation of the basic necessities of life, with restrictions on food intake, poor health conditions and high morbidity rates, which negatively influence children's growth potential.^56^^,^^57^^,^^58^ Thus, the Bolsa Família program, which is based on fulfillment of healthcare-related conditional factors, goes beyond income transfer to inclusion of the population involved, in healthcare actions and primary healthcare services that minimize inequities, thereby adding quality consistent with the potential relating to nutritional status, as explained above.^52^

In relation to the context of public healthcare services, two situations have been suggested: i) there may be greater use of these services among disabled people or those suffering from diseases, who have higher predisposition towards nutritional deficiencies; and ii) healthcare may have a positive effect on nutritional status,^59^^,^^60^ which cannot be indicated through the results from the meta-analysis shown in this work. In this regard, it is important to highlight that the lack of effect of the Bolsa Família program on indicators relating to children's health may be related to the characteristics of program implementation.^61^ This situation indicates that inclusion of input from nutritionists and adequacy of food and nutrition actions are important conditions relating to compliance with the principles of comprehensiveness, universality and problem resolution within healthcare.^62^^,^^63^

Regarding overweight/obesity, the results from this study did not indicate any odds ratios indicative of risk or protection in relation to the population-based rates that were used as a reference, for any of the epidemiological scenarios considered. These data may indicate that the distribution of overweight/obesity is equitable among Brazilian children, without any differences in vulnerability. These findings are consistent with the prevalence of overweight/obesity of 5.4% that was found among children in *quilombo* communities in Brazil,^48^ as well as the results that systematize this problem among Brazilian children,^64^^,^^65^ including those institutionalized in daycare centers.^66^ In addition to the high frequency of overweight/obesity, the findings from this review provide a warning in relation to progressive increases in rates,^64^^,^^65^ equitable distribution across social classes^64^ and the nutritional transition process and its related factors, in determining the problem.^64^^,^^65^^,^^66^ Thus, there is a need for preventive measures aimed towards the coexistence of nutritional deficits and excesses, with a view towards ensuring food and nutritional security and the human right to food.

The results presented here show that, despite the undoubted importance of population-based studies nationwide, they do not discriminate between specific situations and, thus, differences prevail according to different epidemiological contexts. In turn, analyses that enable such differentiation make it viable to ascertain the distribution of nutritional disorders and social inequalities within healthcare and therefore to identify the need for specific and differentiated nutritional and health actions.^57^^,^^67^

This review, in particular, had some limitations, particularly its inclusion of articles identified in just three bibliographic databases, which restricted the analysis spectrum. Nevertheless, consultation of the reference lists of articles previously included in the review was adopted as a methodological strategy that could minimize this limitation. Despite this restriction, the relevance of the results needs to be highlighted, considering their exceptional nature, given that sources characterized by their quality were used in this review. In this regard, the results presented here demonstrate the challenge faced in developing research and public policies to deal with a nutritional problem among Brazilian children that encompasses stunting in marginalized segments and overweight/obesity in all population groups.

## CONCLUSION

The results from this study show the social determinants of stunting among Brazilian children. Its prevalence in populations in situations of social inequity and in populations that use public healthcare services or social registers remains a matter for concern. These populations also present significant overweight/obesity rates. Thus, there is need for appropriate public policies focusing on these realities, while recognizing the possible obstacles implicit in structures and processes that might compromise the effectiveness of these actions.
